# Supplementary material for: Image information optimization processing based on fractional order differentiation and WT algorithm
Source: PLoS One. 2025 May 23;20(5):e0324392. doi: 10.1371/journal.pone.0324392 (PMC12101702; doi:10.1371/journal.pone.0324392)
Supplement: Attachment — Submitted filename: Response to comments.doc [file pone.0324392.s002.doc]

Review Comments to the Author

Please use the space provided to explain your answers to the questions above. You may also include additional comments for the author, including concerns about dual publication, research ethics, or publication ethics. (Please upload your review as an attachment if it exceeds 20,000 characters)

Reviewer #1: Paper is nicely written

All experimental analysis is done nicely

Paper is of good quality. Literature is done in detailed.

Methodology is explained in details. Results shown in graphical as well as tabular form.

Reply: Thank you very much for your kind evaluation.

Reviewer #2:

The proposed method, combining fractional order differentiation (FOD) and wavelet transform (WT), is innovative and technically sound. The integration of edge detection and image fusion techniques is well-justified and aligns with current challenges in complex image processing.

However, further discussion on the method's generalizability to other datasets and scenarios would enhance its impact.

Reply: Thank you very much for your comments. In this study, the LFW and Kaggle PCB Defects datasets were used to train the algorithm, and the Rice Image dataset was used as an independent dataset to test the generalization performance of the algorithm. Please see details as follows:

The datasets used in the experiment include Labeled Faces in the Wild (LFW) dataset, Kaggle PCB Defects dataset, and Rice Image dataset. To test the generalization performance of the proposed algorithm, the LFW dataset and Kaggle PCB Defects dataset will be used for training, while the Rice Image dataset will be used as an independent dataset to test the algorithm's performance.

Overall, the performance of the algorithm proposed in the study on independent datasets is not significantly different from that of the LFW dataset and Kaggle PCB Defects dataset, indicating that the model has good generalization performance.

The results are adequately supported by relevant metrics such as precision, recall, and information entropy. While these provide a strong foundation, adding statistical significance testing (e.g., t-tests or ANOVA) would improve the rigour of your analysis.

Reply: Thank you very much for your suggestion. The statistical significance test has been added. For example:

Table 1. Quantitative analysis results of edge detection

| Image Name | B/A of PA | B/A of RA | B/A of LOG-A | B/A of CA | B/A of SA |
| --- | --- | --- | --- | --- | --- |
| Experiment 1 | 0.0915 | 0.0880 | 0.0652 | 0.0248* | 0.0143* |
| Experiment 2 | 0.2934* | 0.3039* | 0.0868* | 0.0270* | 0.0219* |
| Experiment 3 | 0.0270* | 0.0222* | 0.0224* | 0.0152* | 0.0146* |

Note: * indicates that *p* <0.05.

While the manuscript is mostly clear, specific phrases and sentences are awkwardly structured or repetitive. For instance:

"The highest recall rate was 80%, the minimum information entropy was 3.13..." could be simplified to "The method achieved an 80% recall rate and a minimum information entropy of 3.13."

Reply: Thank you very much for your comments. The full text has been checked and complex sentences have been simplified. For example, the sentence “The highest recall rate was 80%, the minimum information entropy was 3.13...” was changed to “The method achieved an 80% recall rate and a minimum information entropy of 3.13.”

Avoid redundancy, such as "the study proposed method edge detection by the study."

Reply: Thank you very much for your comments. The sentence was changed to “The precision of the proposed method edge detection by the study...”

Ensure consistency in technical terminology, and avoid using acronyms without proper explanation.

Reply: Thank you for your suggestion, the abbreviation form has been added as follows:

List of Abbreviations

FRWT: Fractional Wavelet Transform

WT: Wavelet Transform

FOD: Fractional order derivative

FO: Fractional order

SMC: Sliding mode controller

LF: Low frequency

TF: Temporal frequency

DWT: Discrete wavelet transform

RL: Riemann-Liouville

G-L: Grimwald-Letnikov

ED: Edge detection

RO: Roberts Operators

PO: Prewitt Operators

SO: Sobel Operators

LOG-O: LOG Operators

CA: Canny algorithm

GF: Gaussian filtering

GAV: Gradient amplitude value

PP: Pixel point

CO: Canny Operators

HF: High frequency

CWT: Continuous wavelet transform

OS: Original signal

MV: Mean value

var: variance

cov: Covariance

SA: Sobel algorithm

RA: Roberts algorithm

LOG-A: LOG algorithm

PA: Prewitt algorithm

LFW: Labeled Faces in the Wild

The figures and tables effectively support the narrative but could benefit from more explicit captions that summarize their findings.

Reply: Thanks for your advice. The figures and tables titles have been modified as follows:

Figure 8. Image edge detection MSE with accuracy for different operators

Figure 9. Information entropy and recall of image edge detection by different operators

Table 4. The image fusion quality of six algorithm

Figure 10. Average gradient and information entropy for different image fusion methods

Figure 11. The spatial frequency and mutual information of the different image fusion methods

Ensure all axes and labels are legible, particularly in performance comparison graphs (e.g., Figures 8–11).

Reply: Thanks for your advice, the figures has been modified as follows:

Figure 8. Image edge detection MSE with accuracy for different operators

Figure 9. Information entropy and recall of image edge detection by different operators

Figure 10. Average gradient and information entropy for different image fusion methods

Figure 11. The spatial frequency and mutual information of the different image fusion methods

The discussion section should expand on how the proposed method could be optimized or applied to other domains like video processing or real-time applications.

Reply: The discussion section is revised according to your kind suggestions. Please see details as follows:

The study proposed a method for optimizing image information processing that is based on FOD and WT algorithms. This method has demonstrated effectiveness in the domains of image edge detection and image fusion. Through experimental verification, this method performs well in image edge detection and image fusion, especially in key indicators such as detection accuracy, information entropy, recall rate, and mean square error (MSE), which are superior to traditional methods. X. Luo et al. proposed a fusion method based on quaternion WT and feature level copula model for image fusion problems. This method was developed to avoid the visual quality degradation of images caused by false information, as compared with other methods [40]. D. Agrawal et al. proposed an image fusion method based on DWT. This approach was developed to improve the visibility and quantitative performance of fused images, as compared with other methods [41]. However, despite significant achievements, there are still some potential optimization directions and application extensions worth further exploration. To further improve the performance of this method and apply it to a wider range of fields, such as video processing and real-time applications. In real-time applications, such as surveillance systems and autonomous driving, the real-time and accuracy of image processing are crucial. To achieve real-time processing, the methods proposed in the research can be simplified and optimized. For example, reducing the number of layers in wavelet decomposition or using fast fractional order differentiation algorithms. In addition, the combination of hardware acceleration technology can further improve real-time processing capabilities. Meanwhile, to adapt to the constantly changing environment in real-time applications, online learning mechanisms can be introduced to enable algorithms to dynamically adjust parameters to adapt to new image features.

Address potential limitations of the method, such as computational efficiency or dependency on parameter tuning.

Reply: Thanks for your advice, the potential limitations of the method and the solutions have been added as follows:

Due to the fact that only 2D and grayscale images were considered when constructing the proposed algorithm, its ability to handle higher dimensional or more complex image data is not yet clear. In view of this, it is considered to introduce directional fractional wavelet transform in the future to improve the algorithm's ability to process high-dimensional images.
