# Supplementary material for: Image information optimization processing based on fractional order differentiation and WT algorithm
Source: PLoS One. 2025 May 23;20(5):e0324392. doi: 10.1371/journal.pone.0324392 (PMC12101702; doi:10.1371/journal.pone.0324392)
Supplement: Attachment — Submitted filename: Response to reviewers comments.docx [file pone.0324392.s003.docx]

Additional Editor Comments (if provided):

Please revise the paper based on reviewer comments. Also consider the editor comments below.

1. A more detailed literature on the study should be added at the end of the Related works section. After this process, the difference of this study from the literature should be interpreted more clearly.

Reply: Thank you very much for your advice, a summary about the literature has been added at the end of the Related works section, and the difference of this study from the literature has been explained. Please see details as follows:

In summary, the above literature demonstrates the powerful application potential and advantages of fractional calculus and WT techniques in multiple fields. These studies have achieved significant results in improving system performance, enhancing robustness, and solving specific problems. However, there are still some challenges and limitations in terms of implementation complexity, computational cost, model dependency, dynamic response performance, long-term stability, and adaptability to complex environments, which need to be addressed and optimized in further research in the future. The proposed method combines fractional order differentiation and wavelet transform algorithms, which can effectively extract image edge information while preserving weak edge information in smooth areas. This method introduces fractional order differentiation, allowing the algorithm to adjust the differentiation order more flexibly and thus more finely control the extraction of local features in the image.

2. Although there are many different methods that can be used for image edge detection in the literature, why a method based on feature fractional gradient operator was used in this study and its originality should be stated more clearly.

Reply: Thank you for your suggestion. The reasons and originality of the fractional gradient operator have been added as follows:

The fractional gradient operator can finely control the local feature extraction of signals or images by adjusting the differential order, thereby achieving optimal performance in different application scenarios. Compared with integer order differentiation, fractional gradient operators can better capture subtle changes in signals, especially when dealing with complex nonlinear systems. Secondly, the fractional gradient operator also has memory, that is, it not only considers the value of the current point, but also considers the values of all past points. In addition, the fractional gradient operator can more effectively suppress noise while extracting signal features. This is because the fractional gradient operator can balance the smoothness of the signal and the accuracy of feature extraction by adjusting the order, thereby preserving important features of the signal while removing noise.

3. The results obtained from the scope of the study and the metric types seem to be at an appropriate level when compared to the literature.

Reply: Thank you for your opinion. Compared with other literatures, the edge detection accuracy, execution efficiency and image fusion quality of this study method are significantly improved. Please see the experimental results as follows:

Table 3. B/A of different edge detection algorithms

| Image Name | TBSWT-FM | ICO | Our Method |
| --- | --- | --- | --- |
| Experiment 1 | 0.036* | 0.041* | 0.015* |
| Experiment 2 | 0.042* | 0.039* | 0.022* |
| Experiment 3 | 0.033* | 0.028* | 0.015* |

Table 4. The image fusion quality of six algorithm

| Fusion method | P=0.4 | | | P=0.5 | | | Reference |
| --- | --- | --- | --- | --- | --- | --- | --- |
|  | MI | QAB/F | SF | MI | QAB/F | SF |  |
| FRWT | 5.018 | 0.698* | 12.659* | 5.121* | 0.741* | 9.098* | / |
| NSCT | 4.923 | 0.681 | 9.033* | 4.814* | 0.675* | 7.976* | Lawrance N et al [37] |
| WT | 4.823 | 0.611* | 8.620* | 4.743* | 0.654* | 7.688* | Pramanik S. et al [31] |
| DWT | 4.767 | 0.602* | 7.598* | 4.533* | 0.520* | 5.983* | R. Ahmadian et al [33] |
| PNCC | 4.823 | 0.611* | 8.620* | 4.743* | 0.654* | 7.688* | KP B et al [38] |
| L_P | 4.767 | 0.602* | 7.598* | 4.533* | 0.520* | 5.983* | Song M et al [39] |

4. The steps for implementing guided filtering are clearly stated and clearly reveal the quality of the study.

Reply: Thank you very much for your comments. As shown in Figure 6, firstly, the boxFilter filter is used to complete the correlation coefficient parameter (CCP). The mean value (MV) includes autocorrelation MV, cross-correlation MV, bootstrap image MV, and the original image MV that needs to be filtered. CCP calculates based on MV, including autocorrelation variance (var) and cross-correlation covariance (cov). Secondly, calculate the coefficients of the window linear transformation parameters a and b. The MV of parameters a and b is calculated according to the formula. Finally, use these parameters to obtain the output image of the bootstrap filter.

5. The reason for the dataset preferences used in the study and their places in the literature compared to other datasets in the literature should be stated more clearly.

Reply: Thank you very much for your suggestion, the reasons for using the data set have been explained as follows:

Considering the diversity and representativeness of the dataset, as well as the comprehensiveness of performance evaluation, the selected datasets for the experiment include Labeled Faces in the Wild (LFW) dataset, Kaggle PCB defect dataset, and Rice image dataset. Compared to the datasets used in other literature, experiments conducted on the aforementioned three datasets can verify the applicability of the algorithm in various practical applications. For example, edge detection of facial images can be applied to facial recognition and expression analysis; edge detection of circuit board images can be used for industrial quality control; edge detection of rice images can be utilized for image analysis in the agricultural field.

The LFW dataset is used to test the edge detection performance of the proposed method, the Kaggle PCB dataset is used to verify the edge detection effect of the research algorithm on images with rich edges, and the Rice Image dataset is used to verify the algorithm's noise resistance performance.

6. The hardware or software (toolbox etc.) used in the study should be stated more clearly and detailed.

Reply: Thank you very much for your comments. The software and hardware used in the experiment are described in detail in section 4.1. Please see detais as follows:

The operating system chosen for the experiment is Windows 11X64, RAM is 16GB, CPU is 12th Gen Intel(R) Core(TM) i7-12700H @ 2.30GHz, the graphics card model is NVIDIA GeForce RTX 3060, and the graphics card memory is 16G, and the software chosen is MATLAB R2018a. FO P takes the values of 0.4 and 0.5, respectively.

Review Comments to the Author

Please use the space provided to explain your answers to the questions above. You may also include additional comments for the author, including concerns about dual publication, research ethics, or publication ethics. (Please upload your review as an attachment if it exceeds 20,000 characters)

Reviewer #3:

The authors have clearly stated the proposed method.

The findings obtained demonstrate the effectiveness of the study.

Up-to-date and sufficient references have been added.

The authors have responded constructively to the comments of other reviewers.

Reply: Thank you very much for your satisfaction with the revisions to this article.

Reviewer #4:

The referee comments for the study titled "Image Information Optimization Processing Based on Fractional Order Differentiation and WT Algorithm" are as follows.

*The purpose of the study should be clearly explained.

Reply: Thank you very much for your comments. The purpose of this study has been explained as follows:

In view of this, in order to improve the quality of multi focus image fusion, avoid the loss of local details and edge artifacts, a method based on wavelet transform (WT) algorithm and fractional order differentiation (FOD) is proposed to optimize the information of medical and satellite images.

*The effect of the proposed method should be shown on sample images.

Reply: Thank you for your suggestion, the image is shown in Figure 10 as follows:

Figure 10 Edge detection effects for different datasets

As shown in Figure 10, compared to other algorithms, the proposed method not only achieves accurate edge detection of ordinary images, but also improves the edge detection effect of images with rich edges. In addition, this method has good resistance to salt and pepper noise, and its noise profile is significantly reduced during the detection process.

*The discussion section should be made more understandable with images and tables.

Reply: Thank you for your advice, the images in the discussion section is shown as follows:

Different image information processing optimization methods are shown in Figure 14.

Figure 14. Different optimization methods for image information processing

As can be seen from Figure 14, compared with literature [40] and literature [41], the image information optimization quality of the proposed method is better, which not only retains the new solution intact, but also basically has no noise interference.
